# Poly-(D,L)-Lactide-ε-Caprolactone-Methacrylate Is a Suitable Scaffold Material for In Vitro Cartilage Regeneration

**DOI:** 10.3390/ijms26125837

**Published:** 2025-06-18

**Authors:** Michelle Sophie Wunderer, Veronika Sparenberg, Christoph Biehl, Klaus Liefeith, Katrin Susanne Lips

**Affiliations:** 1 Experimental Trauma Surgery, Justus-Liebig-University Giessen, Aulweg 128, 35392 Giessen, Germany; veronika.sparenberg@med.uni-giessen.de (V.S.); christoph.biehl@chiru.med.uni-giessen.de (C.B.); 2 Department of Trauma, Hand and Reconstructive Surgery, University Hospital of Giessen-Marburg GmbH, Campus Giessen, Rudolf Buchheim Strasse 7, 35392 Giessen, Germany; 3 Department of Biomaterials, Institute for Bioprocessing and Analytical Measurement Techniques e.V. (iba), Rosenhof, 37308 Heilbad Heiligenstadt, Germany; klaus.liefeith@iba-heiligenstadt.de

**Keywords:** osteochondral, cartilage, chondrogenic, SOX9, mesenchymal stem cells, TRPV4, collagen type II, DMOG, heparin coating, polymer discs

## Abstract

Due to the limited regeneration of cartilage, new implant materials are needed. Biodegradable polymers poly-(D,L)-lactide-ε-caprolactone-methacrylate (LCM) and polyamid-ε-caprolactone-methacrylate (ACM) were recently established and coated with heparin, making them able to prevent blood coagulation and cartilage mineralization. The aim of this study was to analyze the suitability of LCM and ACM alone or coated with heparin (the latter are abbreviated as LCMH and ACMH, respectively) as implant material for cartilage repair. Therefore, mesenchymal stem cells were chondrogenically differentiated in 2D cultures with polymer discs. Differentiation was induced by the supplementation of cell medium with dimethyloxalylglycine, TGF-β, and BMP2. After 5 days, no increase in proinflammatory factors was observed. Cell viability declined on ACM and ACMH discs. During early chondrogenesis, SOX9 expression increased on LCM and LCMH discs, while TRPV4 expression decreased on ACMH discs. At day 20, the level of collagen type II increased on LCM, LCMH, and ACM discs, demonstrating the ability of chondrogenic development on these implants. In summary, coating with heparin showed no advantages compared to pure LCM and ACM. For cartilage repair, LCM is more suitable than ACM in this 2D in vitro model, which needs to be verified by long-term 3D models and in vivo studies.

## 1. Introduction

Osteoarthrosis (OA) affected 527.81 million people worldwide in 2019; this number has increased more than twofold since 1990 and is expected to rise further because of the aging society [1]. Due to its increasing prevalence, OA is the most important joint disease that leads to disability and therefore holds a high social burden [2]. Women are affected more often than men [1]. OA leads to the destruction of the articular cartilage, and during its further progress, the subchondral bone is also affected. Clinical treatment is still challenging and controversial. Allogenic or autologous osteochondral tissue transplantation are promising, but they express several disadvantages, e.g., a risk of contamination, donor site morbidity, limited availability, and high costs [3]. Thus, there is an increasing demand for biocompatible scaffolds that contribute to the self-healing of cartilage by stimulating signaling cascades and cell ingrowth but avoid blood coagulation and the increase in inflammatory factors. Several materials have been used with different methods for scaffold generation, but most of them are not suitable for 3D printing, which, following technological advantages, is the most promising method for producing finely structured pores that meet the requirements of cell ingrowth. Therefore, this study aimed to establish a suitable polymer through the 2D analysis of casted discs. In the future, the best polymer shall be used for the fabrication of osteochondral scaffolds by 3D printing, followed by analysis in 3D cell cultures and an in vivo model.

Articular cartilage has limited self-restoring capacities because of its low cell density, lack of vascularization, and alymphatic nature [3]. Cartilage defects are usually filled with replacement tissue made of fibrocartilage, which contains a high proportion of collagen type I fibers, while hyaline cartilage contains mainly masked collagen type II fibers. The aim of implanting cell-free scaffolds is the ingrowth of chondroblast or mesenchymal stem cells (MSCs) from the sides where healthy cartilage and bone are localized.

MSCs are capable of differentiating into cartilage-synthesizing chondrocytes but also into bone-forming osteoblasts, adipocytes, and several other cell types. A possibility to direct MSCs to the chondrogenic cell fate is the addition of specific supplements into the cell culture medium. Besides drug supplementation, in vitro chondrogenesis can be stimulated by pressure or hypoxia. A low oxygen content of 1–6% instead of 20% oxygen stimulates chondrogenesis [4] via hypoxia-inducible factor 1 subunit alpha (HIF-1α) [5]. Hypoxia can also be chemically induced, e.g., by dimethyloxalylglycine (DMOG) [6]. The combined application of DMOG and parathyroid hormone-related protein (PTHrP) facilitated chondrogenesis and prevented hypertrophy [7]. On the contrary, the application of DMOG to porcine synovial-derived MSCs co-cultured with chondrocytes only resulted in a general upregulation of collagen type II deposition [8]. Recently, Dong et al., 2024 [9], summarized in a literature review that DMOG applied to MSCs stimulates the osteogenic as well as the chondrogenic fate and additionally supports angiogenesis. Thus, the effect of DMOG is still controversial.

The differentiation of MSCs into chondrocytes can be characterized by the serial synthesis and release of matrix proteins. Here, changes in the matrix components transient receptor potential vanilloid cation channel 4 (TRPV4), sex-determining region Y (SRY)-box transcription factor 9 (SOX9), aggrecan (ACAN), hyaluronan and proteoglycan link protein 1 (HAPLN1), runt-related transcription factor 2 (RUNX2), collagen type I, and collagen type II were determined by relative mRNA expression measured by real-time reverse transcriptase polymerase chain reaction (real-time RT-PCR).

TRPV4 is a mechano- and osteosensitive ion channel that is highly expressed in chondrocytes, where its activation leads to an anabolic response with increased matrix production [10]. TRPV4 also activates SOX9, which is the most important regulatory factor in the differentiation of MSCs to early chondrocytes. In cooperation with SOX5 and SOX6, it stimulates the synthesis of collagen type II through the activation of a chondrocyte-specific enhancer [11]. As a structuring proteoglycan of hyaline cartilage, ACAN crosslinks collagen II fibrils, forms aggregates with glycosaminoglycans (GAGs), and binds hyaluronic acid [12], thereby forming a negatively charged structure trapping high amounts of water and positively charged ions. According to this, the basic function of ACAN is its resistance to compressive force which is characteristic of articular cartilage [13]. RUNX2 is required for chondrocyte maturation and involved in the decision whether MSCs are differentiated into an osteoblast or chondrocyte lineage. It is expressed by MSCs and regulates the expression of transcription factor SP7 which directs osteoblast progenitors to immature osteoblasts and suppresses the differentiation of MSCs into chondrocytes. RUNX2 is weakly expressed in proliferating chondrocytes and upregulated in hypertrophic and terminal chondrocytes. Its function is to keep the cells in the proliferation stage and to prevent them from changing into the phenotype of permanent cartilage [14]. In the osteoblast lineage, RUNX2 additionally stimulates the osteogenic differentiation of MSCs into osteoblasts. During late osteogenesis, RUNX2 inhibits the differentiation of mature osteoblasts into osteocytes [15]. As in chondrogenic differentiation, osteoblasts are protected from final differentiation and maintained in a state of proliferation and bone matrix production. Collagen type I is associated with cartilage of a fibrocartilaginous phenotype but also expressed in culture systems during chondrogenic differentiation [16]. In hyaline cartilage, the occurrence of collagen type II predominates over collagen type I. In hyaline articular cartilage, 90–95% of the extracellular matrix is collagen type II, whereas in fibrocartilage, collagen type I is most prominent [17]. In the present study, the detection of collagen type II is demonstrated at the mRNA level by real-time RT-PCR and at the protein level using ELISA.

Recently, tissue engineering has made progress in the generation of scaffolds that permit chondrogenic differentiation. Therefore, scaffold material must be biocompatible and allow MSC adhesion, proliferation, and differentiation. In addition, it should serve as a guide for the body’s own cells to grow into the cartilage defect. The scaffold therefore requires adapted biomechanical and biochemical properties and an architecture that allows the ingrowing cells to maintain their information exchange through cell–cell and cell–matrix contacts. For the generation of such scaffolds, 3D printing methods are most suitable. Recently established polyamid-ε-caprolactone-methacrylate (ACM) and poly-(D,L)-lactide-ε-caprolactone-methacrylate (LCM) can be printed in a 3D structure with a specific pore size [18,19]. The in vitro and in vivo biocompatibility of LCM has already been shown in human MSCs and a murine intracalvarial defect model [20,21]. LCM consists of polyesters, which have easily adjustable physical and chemical properties [18]. During the degradation of polylactate-containing materials, lactic acid is released, so the local pH value is reduced during acetolysis [22]. Due to the slow degradation of approximately 10–30% of the dry mass after 80 days at 37 °C, as shown by Felfel et al. in 2016, the immunological reactions of LCM cannot be ruled out by short-term analyses [23]. The degradation of ACM produces the non-toxic monomer caprolactone and the amino acids alanine and glycine [19]. The degradation rate is adjusted by the concentration of caprolactone. Thus, it is possible to adapt material degradation to cartilage regeneration. In addition, the degeneration products should not lead to an immunological foreign body reaction and an increase in proinflammatory factors. Inflammatory cytokines such as tumor necrosis factor-α (TNF-α), interleukin (IL)-1, and IL-6 induce chondrocyte death and the breakdown of the cartilage matrix, enhance the synthesis of proteolytic enzymes, and inhibit the production of collagen type II and GAGs. IL-6 is produced by, e.g., M1 macrophages, monocytes, B cells, lymphoid cells, and endothelial cells, as well as by chondrocytes and MSCs. It has essential functions for the innate and adaptive immune system and increases the inflammatory response by itself but also through the stimulation of TNF-α and IL-1 and the inhibition of anti-inflammatory factors. It is considered as the key cytokine of cartilage destruction [24]. In addition, it enhances osteoclast development and the degradation of the subchondral bone, which makes it extremely relevant for the establishment of osteochondral implants.

Articular cartilage consists of a non-mineralized portion towards the joint and a mineralized layer at the transition to the subchondral bone. The mineralized cartilage is calcified but not ossified as the subchondral bone. The small, calcified cartilage layer builds a biological barrier that is necessary for the proper formation of the non-calcified hyaline cartilage. The glycosaminoglycan heparin has the ability to prevent cartilage mineralization [25]. In addition, heparin is well known for its anticoagulation effect, and it is also able to inhibit angiogenesis and inflammation [26]. Thus, the coating of scaffolds with heparin seems to be promising. Here, we compared pure ACM and LCM discs with heparin-coated ones for their compatibility as future implant materials for the production of porous scaffolds for chondrogenic differentiation, which is one of the key processes during cartilage repair and the healing of osteochondral defects. To address this issue, we established a 2D in vitro system of chondrogenesis and evaluated the new polymer materials using real-time RT-PCR, light microscopy, collagen type II and IL-6 ELISA, and an MTT assay to determine material suitability and cellular viability.

## 2. Results

### 2.1. Viability and Metabolic Activity

**Figure 1:**
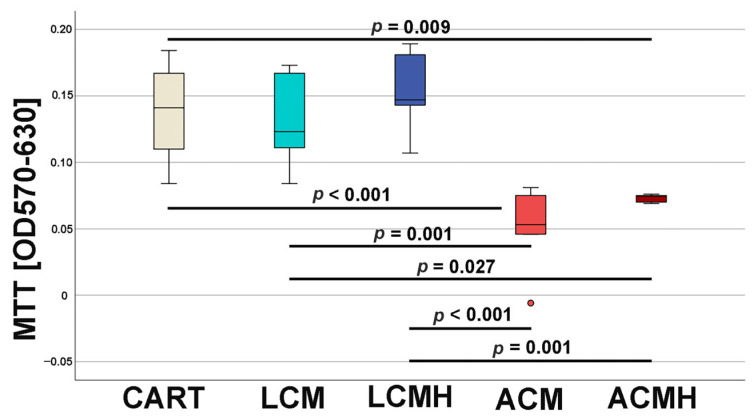
MTT assay for the determination of metabolic activity and cellular viability in direct contact with material discs. CART: positive control, cells incubated in chondrogenic differentiation medium without material. Data are presented as box plots with the median being indicated by a solid line within the box. The box contains 50% of the data, with the length of the box being the interquartile range, the distance between the 3rd and 1st quartile. The 3rd quartile or the upper edge of the box represents 75% of the data that are ≤ this value. The lower limit of the box represents the 1st quartile, and thus, 25% of the data below this range are ≤ this value. The whiskers show minimum and maximum data that are below 1.5 times the interquartile range. Outliers above 1.5 times and below 3 times the interquartile range are represented as circles. ANOVA followed by post hoc test were used for statistical analysis (n = 6).

The MTT assay was used to determine the metabolic activity and viability of the cells (n = 6). The viability was significantly decreased in ACM and ACMH compared to LCM and LCMH. LCM and LCMH showed no significant differences to the positive control (CART), whereas ACM and ACMH were significantly reduced (Figure 1 and Table S1). Hence, cells in ACM and ACMH resulted in a restriction in metabolic activity and cellular viability during early chondrogenic differentiation, and therefore LCM and LCMH are more suitable as chondrogenic substitution materials according to the MTT assay.

### 2.2. IL-6 ELISA

**Figure 2:**
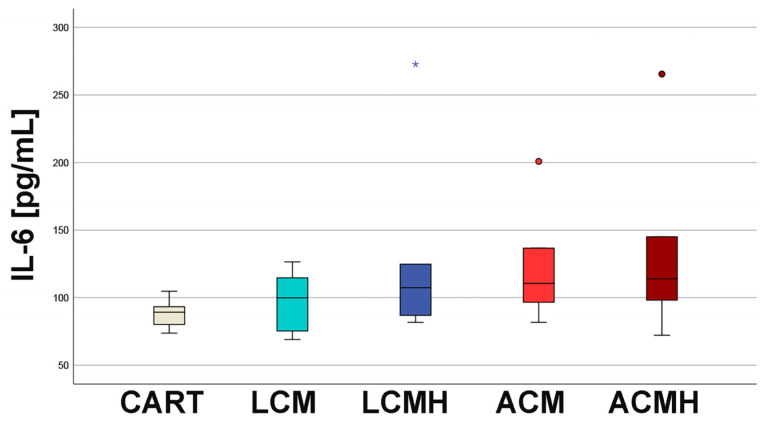
The IL-6 ELISA revealed no increase in inflammation. A Kruskal–Wallis test was used for statistical analysis (*p* = 0.289, n = 6). Outliers above 1.5 times and below 3 times the interquartile range are represented as circles, and extreme values above 3 times the interquartile range are plotted as asterisks.

Nonspecific immune reactions are triggered by interleukin-6 (IL-6). As a proinflammatory cytokine, it activates acute-phase proteins [24]. No significant differences were detected between the material groups and CART, which was the differentiation control without material (Figure 2 and Table S2). In addition, the IL-6 ELISA was verified by the controls. The assay itself was controlled by a standard of the manufacturer that showed the measurement of IL-6. Additionally, MSCs stimulated with LPS for 1, 4, and 24 h achieved values of 1660, 2186, and 2259 pg/mL, which were much higher than those of any of the sample groups. Thus, the IL-6 ELISA worked properly, but there was no detectable increase in the proinflammatory marker IL-6 in any of the samples, which is an important prerequisite for the further use of the polymers.

### 2.3. TNF-α ELISA

**Figure 3:**
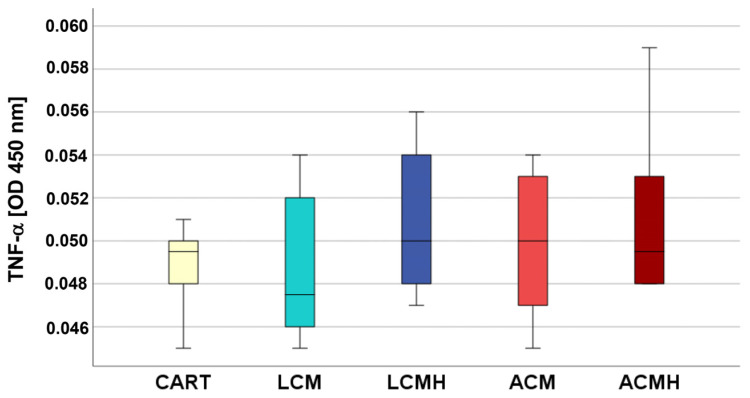
The TNF-α ELISA showed no proinflammatory reaction in the test samples. Statistical analysis was performed using a Kruskal–Wallis test that did not result in significant differences between the groups (*p* = 0.698, n = 6).

Relevant amounts of the proinflammatory factor TNF-α were not measured in the material groups or in the CART. No significant differences were detected between the material groups (Figure 3, Table S3). The ELISA was controlled by positive controls: (a) the internal positive controls of the ELISA kit and (b) MSCs that were stimulated with LPS. Both positive controls revealed a clear signal. Thus, the TNF-α ELISA verified the absence of a proinflammatory reaction of the MSCs cultured on the polymers.

### 2.4. IL-1β ELISA

**Figure 4:**
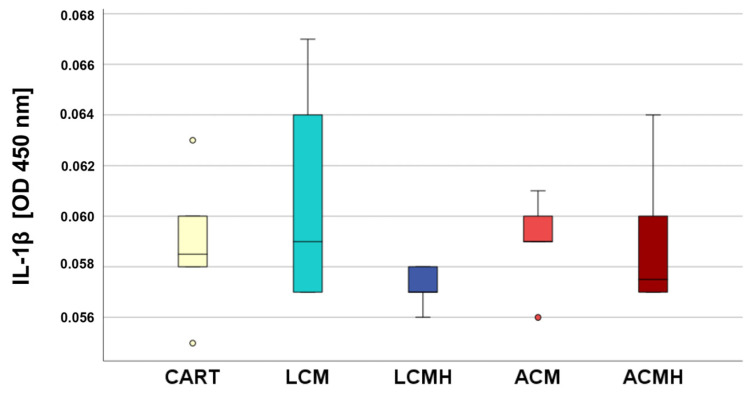
No proinflammatory reaction was detected by the IL-1β ELISA. The statistical analysis with the Kruskal–Wallis test revealed non-significant differences between the groups (*p* = 0.291, n = 6). Outliers above 1.5 times and below 3 times the interquartile range are represented as circles.

The IL-1β ELISA was used to verify the results of the IL-6 ELISA. Small amounts of IL-1β were detected in the material groups as well as in the CART. No significant differences were found between the groups (Figure 4, Table S4). The internal control of the ELISA kit (Invitrogen, Thermo Fisher Scientific, Waltham, MA, USA) and MSCs stimulated with LPS were used as the positive controls that showed a distinct IL-1β presence. Thus, the results of IL-1β, in addition to TNF-α, verified the absence of a proinflammatory reaction of MSCs on polymer implants of LCM and ACM with and without a heparin coating.

### 2.5. Relative TRPV4 mRNA Expression

**Figure 5:**
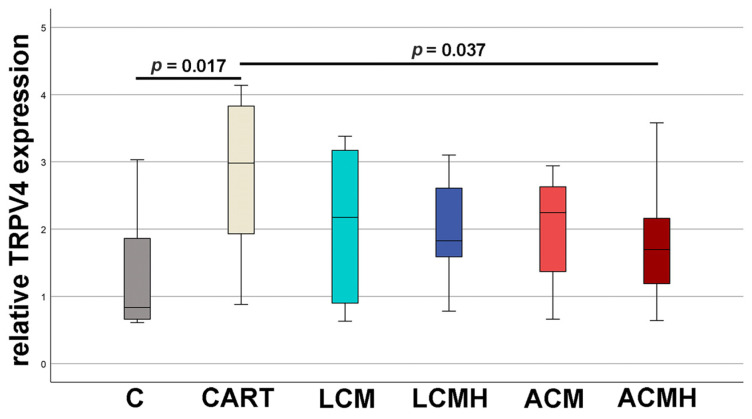
Relative TRPV4 mRNA expression. C: control cells without chondrogenic medium. CART: cell cultured in chondrogenic medium without material. Values were normalized to samples of day (d) 0. Statistical analysis was performed using paired-samples *t*-tests. The *p*-values not shown in the figure were all *p* > 0.05 (n = 6).

Transient receptor potential vanilloid cation channel 4 (TRPV4) is one of the earliest markers of the chondrogenic fate. At day 5, TRPV4 mRNA expression was increased significantly in MSCs incubated in chondrogenic differentiation medium compared to cells in control medium without chondrogenic stimulation (*p* = 0.017; n = 6). Cells incubated on the discs did not reveal an upregulation of TRPV4 expression compared to the control (Figure 5 and Table S5). Instead, a significant decrease in TRPV4 expression was detected for ACMH. The MSCs on the other discs did not show any regulation. Thus, the earliest chondrogenic differentiation of MSCs was delayed on all the discs, but it was worst on the ACMH plates. Thus, the coating with heparin seems not to be suitable for the chondrogenic differentiation of MSCs.

### 2.6. Relative SOX9 mRNA Expression

**Figure 6:**
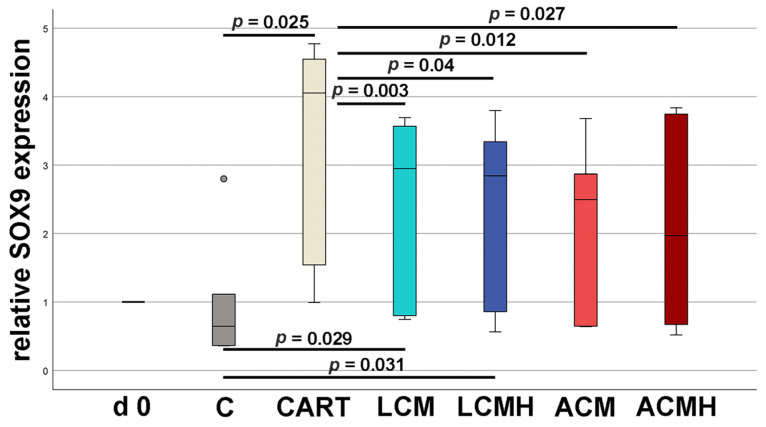
Relative SOX9 mRNA expression. Statistical analysis was performed using paired-samples *t*-tests. The *p*-values not shown in the figure were all *p* > 0.05 (n = 6). Values were normalized to samples of day (d) 0. The small circle represents an outlier.

In addition, SOX9 inhibits the differentiation of early chondrocytes into terminal hypertrophic chondrocytes that no longer synthesize cartilage matrix [27]. Our results showed an increased expression of SOX9 mRNA in cells on LCM (*p* = 0.029) and LCMH (*p* = 0.031) discs compared to the negative control without chondrogenic differentiation medium (Figure 6 and Table S5). Compared to the positive control (CART) with chondrogenic supplementation in the medium, all analyzed materials expressed significantly reduced SOX9 mRNA. Thus, the polymers suppressed the expression of SOX9. However, the LCM materials were more supportive than the ACM ones.

### 2.7. Relative Aggrecan mRNA Expression

**Figure 7:**
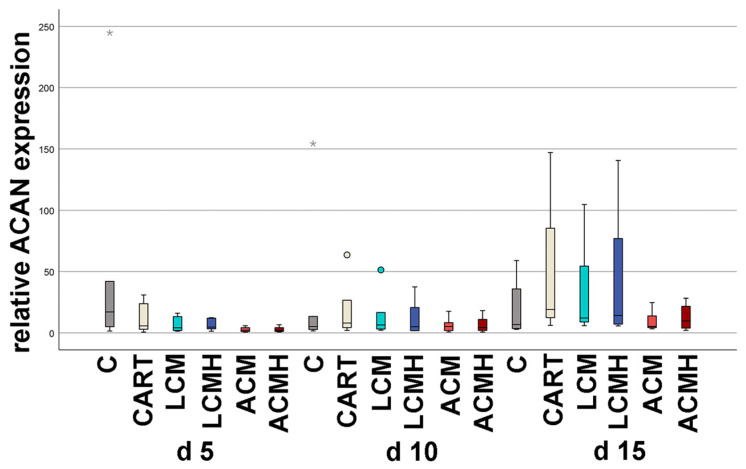
Relative ACAN mRNA expression. The Friedman test with *p* < 0.001, followed by Bonferroni correction, revealed significant differences between some of the groups (d 5 ACM vs. d 15 LCMH, *p* = 0.024; d 5 ACM vs. d 15 LCM, *p* = 0.024; d 5 ACMH vs. d 15 CART) that were not of biological relevance (n = 6). Outliers above 1.5 times and below 3 times the interquartile range are represented as circles, and extreme values above 3 times the interquartile range are plotted as asterisks.

No significant differences in biological relevance were found in ACAN mRNA expression (Figure 7 and Table S6). From day 10 to day 15, ACAN mRNA increased in all material groups as well as in the control groups. Mature cartilage cells seem to be present only at day 15. At day 15, the median values of 5.27 for ACM and 9.8 for ACMH were remarkable low compared to the median values of 12.17 for LCM, 14.27 for LCMH, and 19.09 for the chondrogenic control. Thus, the materials reduced the ACAN expression but did not inhibit it. LCM and LCMH achieved the highest expression among the material groups. However, there was a high variance among the groups.

### 2.8. Relative HAPLN1 mRNA Expression

Hyaluronan and proteoglycan link protein 1 (HAPLN1), together with hyaluronic acid, stabilizes proteoglycans of the extracellular matrix, especially aggrecan [28]. In pluripotent cells and the murine osteoblast progenitor cell line MCT3T E1, HAPLN1, in combination with BMP4, promoted osteogenic differentiation [29].

**Figure 8:**
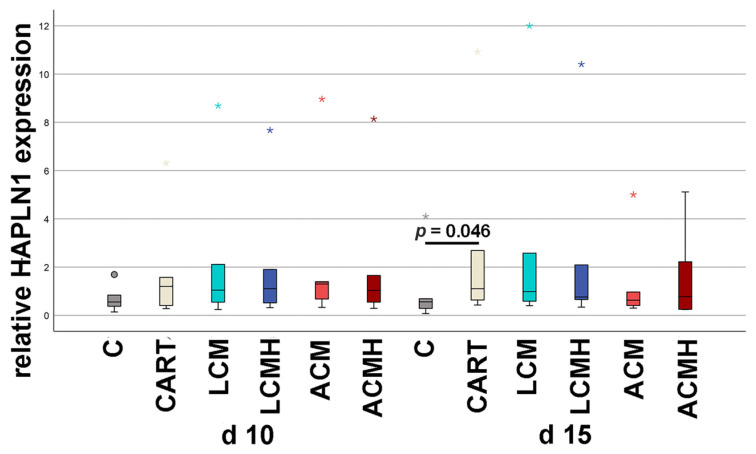
Relative HAPLN1 mRNA expression. Statistical analysis with a Friedman test resulted in *p* = 0.004 (n = 6). The subgroups C and CART at day 15 were subsequently analyzed with a Wilcoxon test (*p* = 0.046). Outliers above 1.5 times and below 3 times the interquartile range are represented as circles, and extreme values above 3 times the interquartile range are plotted as asterisks.

HAPLN1 expression was analyzed at days 10 and 15 of incubation (Figure 8 and Table S6). No significant differences were detected between the different material groups. HAPLN1 expression was generally relatively low apart from a few outliers that are labeled in the graph by small asterisks. A significant increase was only measured at day 15 in the chondrogenic control (CART) compared to the negative control without chondrogenic medium (C). The level of expression of the chondrogenic control (CART) was similar to that of the material groups. Thus, the materials did not inhibit chondrogenic differentiation.

### 2.9. Relative RUNX2 mRNA Expression

**Figure 9:**
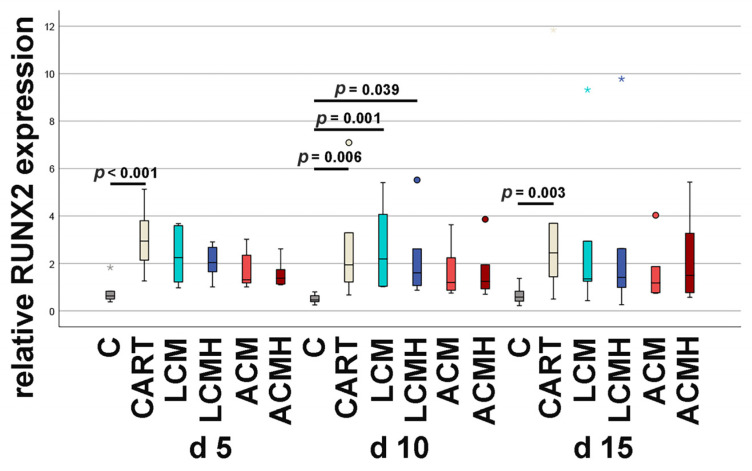
Relative RUNX2 mRNA expression. A Friedman test with subsequent Bonferroni correction was used for statistical analysis (*p* < 0.001; n = 6). The significant and relevant differences are marked in the figure. All other comparisons were either not relevant or resulted in *p* > 0.05. Outliers above 1.5 times and below 3 times the interquartile range are represented as circles, and extreme values above 3 times the interquartile range are plotted as asterisks.

An upregulation of RUNX2 mRNA expression was detected in the positive control (CART, with chondrogenic supplemented medium) compared to the negative control (C, without chondrogenic supplements) at days 5, 10, and 15. A significant regulation of the samples with material was only measured at day 10. The LCM and LCMH groups were significantly increased compared to the negative control (Figure 9 and Table S7). Thus, the materials mostly caused RUNX2 expression to decrease. The upregulation of RUNX2 might extend the proliferation of the progenitors and prevent their differentiation into terminal chondrocytes.

### 2.10. Relative Collagen Type I mRNA Expression

**Figure 10:**
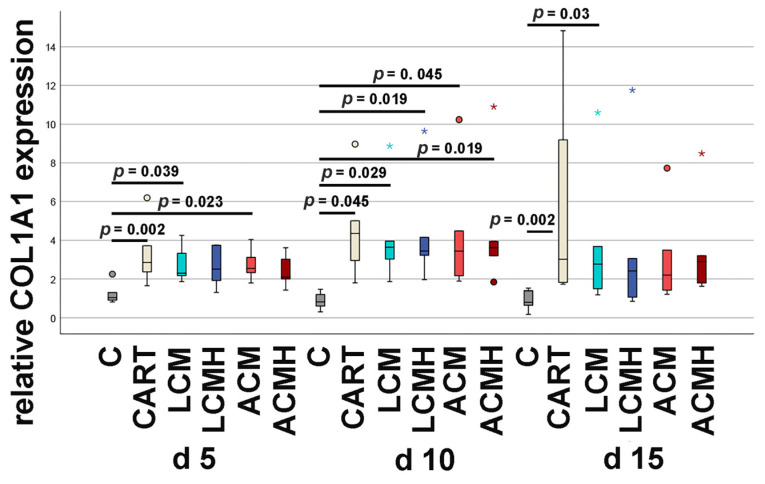
Relative COL1A1 mRNA expression. Statistical analysis was performed using a Friedman test, followed by Bonferroni correction. The relevant significances are marked in the figure. All other comparisons were either not relevant or resulted in *p* > 0.05 (n = 6). Outliers are represented as circles, and extreme values are plotted as asterisks.

In the present study, the expression of collagen type I was increased in the positive controls (CART) compared to the negative control without chondrogenic supplements (Figure 10 and Table S7). An increase in the collagen type I mRNA expression was measured at days 5, 10, and 15 in the positive control (CART). In addition to CART, an upregulation of collagen type I expression was also detected in all material groups at day 10 compared to the negative control (C). At day 5, a higher collagen type I level was only measured for LCM and ACM specimens but not for the heparin-coated plates. At day 15, only LCM specimens showed a significant increase in collagen type I.

### 2.11. Relative Collagen Type II mRNA Expression

**Figure 11:**
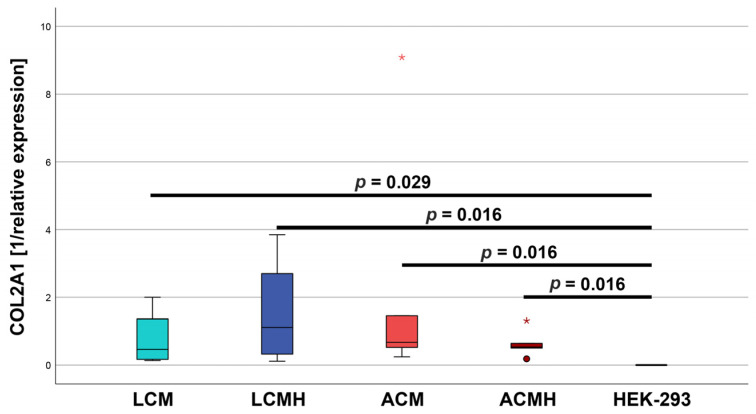
Relative COL2A1 mRNA expression calculated as 1/relative expression. Statistical analysis was performed using a Kruskal–Wallis test, followed by a Mann–Whitney test. The relevant significances are marked in the figure. All other comparisons resulted in *p* > 0.05 (n = 5). Outliers are represented as circles, and extreme values are plotted as asterisks.

At day 15, the relative expression of collagen type II was measured and statistically analyzed using a Kruskal–Wallis test, followed by a Mann–Whitney U-test. For the box plot, the values were calculated as 1 divided by the relative expression (1/2^−ΔΔCT^; Figure 11) because of the high differences between the positive control HEK-293 and the test samples. The HEK-293 mRNA displayed a mean relative expression of collagen type II of 35,801.81 ± SEM of 5434.45, whereas the means (±SEM) of the test groups were determined to be 3.57 ± 1.61 (LCM), 2.74 ± 1.65 (LCMH), 1.68 ± 0.7 (ACM), and 2.33 ± 0.81 (ACMH). There were no significant differences between the material groups (Figure 11, Table S8). Compared to the positive control HEK-293, the collagen type II expression was significantly decreased in all test samples. The CART showed a mean (±SEM) of 1.23 ± 0.71 (n = 3), and thus, its collagen type II mRNA expression was lower than that of the other test samples.

To sum up the real-time RT-PCR results, no significant differences were determined between the material groups. Compared to the positive control CART, the SOX9 mRNA expression was reduced in all material groups, while TRPV4 expression was only reduced in the ACMH group. For the other targets, significant differences were only found compared to the negative control, except for the collagen type II expression which was only significant compared to the positive control HEK-293.

### 2.12. Synthesis of Collagen Type II

**Figure 12:**
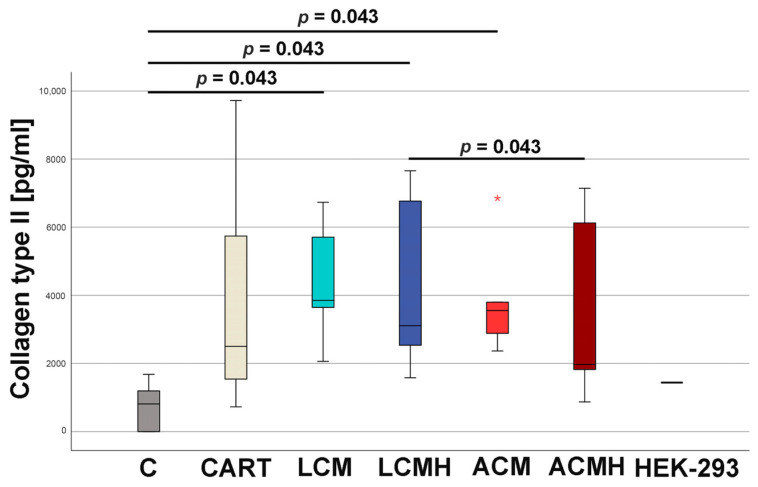
The collagen type II ELISA. Statistical analysis was performed using a Friedman test (*p* = 0.05) followed by a Wilcoxon test, as entered in the figure itself (n = 6). HEK-293 cells were used as a positive control for collagen type II expression. A small asterisk indicates an outlier.

The collagen type II concentration, as detected by ELISA, significantly increased during chondrogenic differentiation in LCM, LCMH, and ACM compared to control cells without chondrogenic medium and without material discs (Figure 12 and Table S9). ACMH was significantly decreased compared to LCMH (*p* = 0.043). The median of all material groups as well as CART was higher than the positive control and cells of the HEK293 cell line, which produce a high amount of collagen type II [30]. Thus, the polymer discs allowed chondrogenic differentiation especially in the LCM group.

## 3. Discussion

This study aimed at investigating the suitability of two different synthetic polymers for the generation of scaffolds for osteochondral regeneration. It was reported that porous 3D scaffolds were most suitable for cartilage regeneration because of enhanced matrix deposition [31,32,33]. However, as a first pilot study, here, we examined material discs and not a porous 3D scaffold. Discs (1 mm in height) allow the clear documentation of the suitability of the material in 2D cell cultures without the overlapping effects of cell proliferation and migration caused by porous scaffolds.

Previously used and approved cartilage implant materials mainly consist of a matrix built with collagen and hyaluronate with short resorption times [34]. In arthritis, catabolic processes predominate and lead to a fast breakdown of these natural polymers. Synthetic polymers offer greater stability and, thus, the necessary time for cartilage formation before the scaffold is degraded [23,35]. The polymers LCM and ACM contain a high proportion of semi-crystalline polycaprolactone, which means that implants made of this material have high biomechanical stiffness and are therefore ideal for cartilage scaffolds implanted into the joints where they should withstand weight loading [19].

At day 5 of chondrogenic differentiation, the viability and metabolic activity of the cells on the materials were investigated by means of an MTT assay. Previous studies measured a higher viability of undifferentiated MSCs on a porous LCM scaffold compared to discs [20]. Thus, when comparing the materials in this study, it was surprising that LCM discs performed better. LCM discs showed no difference to the chondrogenic control CART, while ACM and ACMH discs revealed a significant reduction in cell viability and metabolic activity. Since our study was the first direct comparison of LCM and ACM, the real-time RT-PCR of the early chondrogenic target SOX9 was used for verification. The relative SOX9 expression was upregulated on LCM and LCMH discs but not on ACM and ACMH. Additionally, a significant reduction in collagen type II was measured on ACMH compared to LCMH according to the ELISA. Thus, it was demonstrated with three independent methods that LCM and LCMH are more suitable for the generation of scaffolds for cartilage defect healing compared to ACM and ACMH.

Cell adhesion to synthetic polymers is often reduced compared to that to natural materials because of the low number of adhesion sequences [34]. Thus, coatings are often used to promote cell adhesion. A heparin coating is expected to improve cell adhesion and reduce hemostasis upon contact with blood. Hauptmann et al. (2022), analyzed cell adhesion and the proliferation of undifferentiated MSCs on ACM compared to ACMH by means of immunofluorescence labeling, followed by confocal laser scanning microscopy [19]. They reported reduced proliferation and adhesion on ACMH compared to ACH [19]. These results are in line with our observations for the early chondrogenic differentiation of MSCs on ACMH. The relative mRNA expression of TRPV4 was reduced on ACMH discs but not on ACM. Since the only difference between ACM and ACMH was the heparin coating, it seems that it is responsible for the bad outcome of ACMH. The negative effect of the heparin coating could be caused by the ability of heparin to bind growth factors such as TGF-ß1 and BMP-2 [36]. We supplemented the chondrogenic differentiation medium with TGF-ß1 and BMP-2. Heparin might have absorbed these growth factors, and therefore they were no longer available to stimulate chondrogenic differentiation. This would explain the negative effect we detected for the heparin coating of ACMH. The collagen type II protein synthesis was reduced on ACMH discs compared to LCMH. This difference might be caused by the polymers but not the heparin coating since both polymer discs received the same coating. None of our results revealed significant differences between LCM and LCMH. Thus, the heparin coating did not have any negative or positive influence in combination with LCM in chondrogenic differentiation medium for up to 20 days. This could be due to the slow degradation of LCM described by Felfel et al., 2016 [23], who showed only 10–30% of dry mass loss after 80 days of incubation in PBS at 37 °C. Thus, long-term incubation in chondrogenic medium would be of interest for further studies. At this time, heparin coating can be neglected for LCM, as it has shown no positive or negative effects. The use of heparin coating for ACM must be firmly rejected, as ACM without heparin coating was clearly shown to be more suitable as a material for cartilage implants.

The chondrogenic differentiation of MSCs is still a challenge. Here, it was the basis for the comparison of the four implant discs. Therefore, not only were the materials compared but so was chondrogenic differentiation, assessing the quality of cartilage development. Worldwide, working groups have established protocols for the differentiation of MSCs into chondrocytes. The aim of the differentiation is the synthesis of an extracellular matrix (ECM) with the highest possible content of collagen type II and proteoglycans typical of cartilage such as aggrecan and HAPLN. In general, there are three main differentiation strategies: (i) the addition of growth factors, (ii) the induction of hypoxia, and (iii) the application of mechanical pressure. Here, we used a combination of growth factors and hypoxia that was induced by DMOG. Sathy et al. (2019) reported that MSC-, DMOG-, TGF-ß3-, and BMP-2-loaded hydrogels implanted in mice promoted the formation of a stable cartilaginous tissue with an accumulation of GAGs [31]. DMOG was released for 72 h [31], which is why we applied it for the same time span.

The chondrogenic effect of TGF-β, which leads through the induction of SMAD signaling pathways to the formation of a chondrogenic cell type and inhibited hypertrophy, is well known. The application of TGF-β1 also activates the MAPK pathway by which cellular proliferation, survival, and chondrogenic differentiation are enhanced [37,38]. In pellet cultures, the exposure of TGF-β1 for only 24 h already induced chondrogenic differentiation, as measured by the increased gene expression of SOX9, aggrecan, and collagen type II. In addition, typical osteogenic genes were upregulated, e.g., RUNX2 and collagen type I. To avoid the well-known in vivo enhancement of enchondral ossification by TGF-β1 [39], the usage was limited in time and concentration (10 ng/mL at day 0–5, 7.5 ng/mL at day 5–7, and 5 ng at day 7–17). A dual activity on osteogenic and chondrogenic fate is also known for BMP2 [40]. The combination of TGF-β1 and BMP2 revealed an elevated proteoglycan synthesis [41] and a cartilage-like correlation of collagen type II to type I [42]. Since the long-term application of BMP2 was most promising in an in vivo model [43], BMP2 was added during the whole cell culture period at a concentration of 100 ng/mL. The combination of BMP2 with dexamethasone increased SOX9 expression, while the late chondrogenic targets collagen type II and aggrecan were not enhanced. When dexamethasone was combined with TGF-β1, the chondrogenic differentiation of MSCs was stimulated, whereas the differentiation of synovial fibroblasts into chondrocytes was inhibited [44]. Thus, the cell type also appears to have an influence on chondrogenic differentiation. Often, when articular cartilage is repaired, no hyaline cartilage is created but rather fibrocartilage, which has less compressive elasticity than hyaline cartilage and is, therefore, less suitable for reducing friction and buffering the effect of load-bearing in the joints. However, any replacement tissue is better than destructed hyaline cartilage. Fibrocartilage is characterized by a high amount of collagen type I, whereas 90–95% of the collagen in hyaline articular cartilage belongs to collagen type II [17]. Our results demonstrated that the chondrocytes developed on the polymer discs were able to synthesize collagen type II. However, real-time RT-PCR also showed an upregulation of collagen type I on the implants and the positive control for chondrogenic differentiation (CART). We therefore hypothesize that the selected in vitro system for chondrogenic differentiation, although capable of producing type II collagen, synthesized only a small amount of collagen type II within the available time and instead increased the production of collagen type I. It is therefore possible that the resulting monolayer resembles fibrocartilage rather than hyaline articular cartilage. However, this effect is not due to the polymer discs, as shown by the positive control of chondrogenic differentiation, but rather to the selected in vitro system. We suppose that longer incubation times would have increased the proportion of type II collagen in the ECM but would also have entailed the risk that differences between the material groups would no longer have been noticeable due to advanced cartilage development. However, the length of most in vitro studies of cartilage synthesis using models is between 1 and 4 weeks, as reported in the review of Goldberg et al., 2017 [45]. Therefore, the present study with an in vitro incubation time of 15 days for real-time RT-PCR and 20 days for collagen type II ELISA seems to fit well with the comparative literature. Cai et al. reported in a 3D system the presence of collagen type I in the extracellular matrix (ECM) of the early time points of chondrogenic differentiation when some of the MSCs first started their chondrogenic fate [46]. On the other hand, it has been reported that collagen type I is associated with hypertrophic chondrocytes [47]. During enchondral ossification, chondrocyte hypertrophy together with ECM calcification is a crucial step for the opening of the cartilage matrix by chondroclasts and the concomitant synthesis of the bone matrix directly onto the cartilage matrix by osteoblasts. RUNX2 is a key transcription factor for osteogenic differentiation and required for chondrocyte maturation. It is involved in the decision whether MSCs are differentiated into osteoblasts or a chondrocyte lineage. In proliferating chondrocytes, it is only weakly expressed but upregulated in hypertrophic and terminal chondrocytes where it suppresses the termination of chondrocyte differentiation and re-introduces the cells to a proliferative stage [14]. Here, no significant changes in the relative RUNX2 expression were observed on the implant discs at days 5 and 15. Only at day 10 was RUNX2 mRNA increased on LCM and LCMH discs, which might be caused by residuals of the higher TGF-β1 concentration up to day 7 of incubation. Since RUNX2 mRNA expression decreased at day 15, we suppose that RUNX2 does not represent ossification but chondrogenic differentiation in our setting. Chondrogenic fate is also supported by the fact that collagen type II was detected at mRNA and protein level, even if only in small amounts. At the mRNA level, all test groups expressed significantly less collagen type II mRNA compared to the positive control HEK-293, whereas at the protein level, the median collagen type II concentration increased compared to the HEK-293 collagen type II synthesis. Since the real-time RT-PCR was performed on day 15 and the collagen type II ELISA was performed on day 20 of chondrogenic incubation, we suppose that during this last 5 days of cell culture incubation, the cartilage development increased sharply. The effect of the incubation time on collagen type II synthesis has been described in other publications. However, using 3D cell culture models, hydrogels, and collagen matrices, a significantly earlier peak in collagen type II synthesis was observed than in our study. Jahanbakhsh et al., 2020, described an increase in the collagen type II expression of MSCs encapsulated in different hydrogels from day 7 to day 14 with a slight decline to day 21 [48]. We hypothesize that the delayed synthesis of collagen type II in our model is due to the lack of an additional stimulus such as hydrogels. The polymers failed to provide a similar stimulus for chondrogenic differentiation to that provided by hydrogels in various studies. However, hydrogels lack the biomechanical stability required for the repair of load-bearing joint regions. Future studies could use porous polymer scaffolds whose pores are filled with a hydrogel. This could provide an additional stimulus for collagen type II synthesis.

Our cell culture model for cartilage regeneration is based on the differentiation of human MSCs into chondrocytes synthesizing cartilage matrix. Stenderup et al. (2003) reported that the ability to proliferate and differentiate is reduced in the MSCs of older donors [49]. Our donors ranged from 18 to 90 years of age, which explains the high variation that was found for some of the targets and assays. Despite the high variance, this wide age range of donors was used to represent all age groups affected by cartilage and osteochondral defects. The usage of cell culture passages P6 to P9 might also have negatively influenced the differentiation. It has been shown that higher passages possess increased senescence, which slows the matrix synthesis but increases the expression of proinflammatory factors such as IL-1β, IL-6, and TNF-α [50] which induces matrix degradation [24]. The examination of the cell culture media from day 5 showed no significant differences in terms of IL-1β, IL-6, and TNF-α. This indicates that the materials did not trigger an inflammatory response and were not cytotoxic. In addition, the results from the MTT assay revealed a high metabolic activity on LCM discs that might be correlated with chondroblast proliferation and the high viability of the cells on the polymer discs. Thus, we recommend LCM as a suitable synthetic polymer for bioprinted porous 3D scaffolds.

## 4. Materials and Methods

### 4.1. Polymer Samples

Polymer discs of 1 mm in height and 7 mm in diameter were kindly provided by cooperation partners at the Department of Biomaterials, Institute for Bioprocessing and Analytical Measurement Techniques e.V. (iba).

Discs were generated from biodegradable LCM with a (D,L)-lactide/ε-caprolactone ratio of 8:4 and ACM, with an L-alanine/ε-caprolactone ratio of 2:8. The synthesis of the polymers has been described previously in detail [19,23].

Both polymers were mixed with 2 wt.% of a commercially available UV photoinitiator, IRGACURE^®^ 369 (Sigma Aldrich, Taufkirchen, Germany), filled into a silicone mask with the above-mentioned dimensions and cured under UV light for 9 min using a Vacuum UV Exposure Unit 2 from proMa systro which works at a wavelength of 365 nm and a power of 120 W. Subsequently, the cured plates were washed several times with acetone to remove uncured polymer and unreacted photoinitiator and then transferred in steps of 80:20, 60:40, 50:50, 40:60, and 20:80 from pure acetone to 70% ethanol for storage.

#### Heparin Modification of Polymer Samples

Coating solutions of the respective polyelectrolytes (poly-l-lysin/heparin) were prepared in HEPES/NaCl buffer (25 mM 4-(2-hydroxyethyl)-1-piperazineethanesulfonic acid, 137 mM NaCl, pH 7.4) at a concentration of 1 mg/mL and sterile-filtered (0.2 µm) before use. Automatic film build-up was performed by employing a dipping robot (DR3, Riegler&Kirstein, Postdam, Germany). The robot was equipped with specially designed sample-holding devices that carry up to 40 discs (diameter: 15 mm; thickness: 0.7 mm). The samples were immersed into the respective solutions at a speed of 5 mm/s (dip and withdraw) and left to incubate for 5 and 1 min in the polyelectrolyte and buffer solution, respectively. Ten dipping positions, corresponding to two polyelectrolyte solutions and four wash solutions (HEPES/NaCl buffer) per polyelectrolyte, were programmed. The film construction was carried out automatically, yielding a polyelectrolyte multilayer (PEM) architecture [PLL-HEP]_20_.

**Figure 13:**
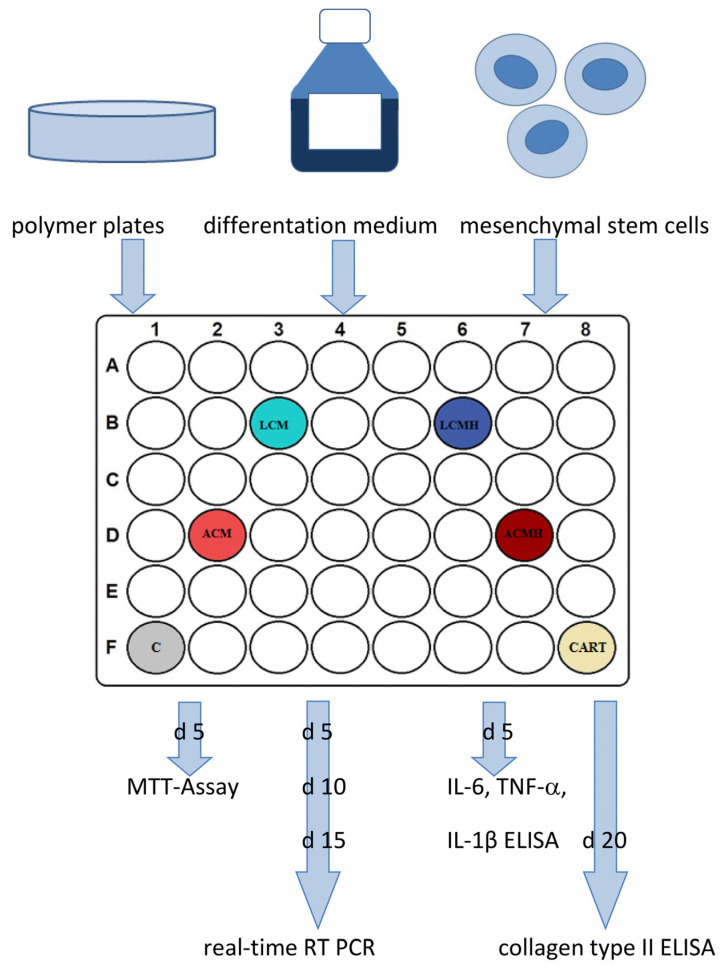
Study design. Polymer discs were washed and incubated with medium before MSCs were seeded and differentiated for 5, 10, 15, and 20 days. The readout parameters were MTT assay, real-time RT-PCR, ELISAs for proinflammatory factors, e.g., IL-6, and collagen type II ELISA.

Before usage, the plates were washed again in 70% fresh ethanol for 24 h and in PBS for 24 h with 3 changes each. Afterwards, the plates were transferred to 48-well plates and covered with medium for 24 h before the cells were seeded to start the experiment. The study design is shown in Figure 13. Technical duplicates were performed for every condition and readout parameter.

### 4.2. Cell Culture

Human MSCs were harvested from the spongy bone of femur heads that were collected during the routine insertion of endoprostheses at the University Hospital of Giessen (Department of Trauma, Hand, and Reconstructive Surgery) after receiving written informed consent from the patients and approval from the local ethic commission (reference number: 74/09), as described previously [20]. In brief, spongy bone was treated with Hibernate A (Gibco, Thermo Fisher Scientific, Dreieich, Germany) containing 10 µg/mL collagenase (Fujifilm Wako, Neuss, Germany) and 0.1 M/mL calcium chloride at 37 °C for 1 h. Digestion was stopped by Hibernate A with 10% fetal bovine serum (FBS, PAN Biotech, Aidenbach, Germany). After erythrocytes were destroyed in lysis buffer for 2 min, the suspension was filtered and washed. Then, fluorescence-activated cell sorting was performed using the Aria II FACS sorter (BD Biosciences, San Jose, CA, USA). The following antibodies were used: (i) PB-conjugated mouse-anti-human CD73 (BioLegend, San Diego, CA, USA), (ii) APC-conjugated mouse-anti-human CD105 (BioLegend), (iii) mouse-anti-human CD31-APC/Cy7 (BioLegend), and (iv) mouse-anti-human CD45-APC/Cy7 (BioLegend). Thus, the sorted MSCs positively bound to CD73 and CD105, whereas they did not bind to CD31 and CD45.

Samples of 6 male donors without pre-existing diseases and bone-influencing medication were involved in the present study. Their mean age was 51.5 years with a range from 18 to 90 years, including one donor each aged 18, 29, 44, 56, 72, and 90 years. This high variance was chosen to reflect the patients’ situation as closely as possible. A separate set of experiments was performed for the cells of each donor and presented as n.

MSCs were bred in a MesenPro RS medium kit (Gibco) with 0.2% gentamicin/amphotericin B, 1% glutamine (Capricorn scientific, Ebsdorfergrund, Germany), and 20% FBS at 37 °C with 6% CO_2_. Chondrogenic differentiation was performed in 48-well plates where 20.000 cells were seeded on the material plates or without material (positive control, chondro). Day 1–5 chondrogenic medium consisted of Dulbecco’s Modified Eagle Medium (DMEM) low glucose (Gibco, Thermo Fisher Scientific Waltham, MA, USA, #11054020) with 10% FBS, 10 ng/mL TGF-β1 (Sigma-Aldrich, Darmstadt, Germany), 100 µM DMOG (Sigma-Aldrich), 100 ng/mL BMP2 (Thermo Fisher Scientific), 1% Insulin–Transferrin–Selenium (ITS+, Merck, Darmstadt, Germany), 50 mg/mL sodium L-ascorbate (Sigma-Aldrich), 40 µg/mL L-proline (Sigma-Aldrich), 1% glutamine, 100 nmol/L dexamethasone (Merck), and 0.2% gentamicin/Amphotericin B. After day 5, DMOG was no longer used as a chondrogenic supplement, and FBS was also reduced to 1%. The concentration of TGF-β1 was reduced to 7.5 ng/mL from day 5 to 7 and to 5 ng/mL from day 7 to 17 of chondrogenic differentiation.

In addition, a negative control (C) was used that received a control medium consisting of DMEM with 10% FBS, 1% glutamine, and 0.2% gentamicin/Amphotericin B up to day 5. Afterwards, the FBS concentration was reduced to 1%.

### 4.3. MTT Assay

The polymer discs situated in a 48-well plate were covered with medium and 20,000 cells per well. At day 5 of cell culture, 50 µL MTT dye (3–4,5-dimethylthizol-2-yl)-2,5-diphenyltetrazoliumbromide, Merck) was added, and the well plate was incubated at 37 °C for 4 h. Afterwards, the supernatant was removed, 500 µL lysis buffer (0.04 N HCl in 2-Propanol) was added, and the well plate was shaken on ice for 10 min. Meanwhile, the color changed from yellow to purple. Then, 200 µL of the supernatant was transferred into a 96-well plate in doublets, and the absorbance was determined with a plate reader (Synergy HT, BioTek Instruments, Winooski, VT, USA) equipped with the software Gen 5 (BioTek Instruments). As background controls, (a) pure lysis buffer (blank value) and (b) material plates without cells in lysis buffer were used. As additional controls, (i) cells in chondrogenic differentiation medium (CART), (ii) cells in control medium without chondrogenic supplements (C), and (iii) cells after 2 h of cell culture were employed. Calculation and statistical analysis were performed after the subtraction of the background controls.

### 4.4. ELISAs for Proinflammatory Factors

MSCs were cultured on the polymer plates for 5 days, the supernatant was collected, and the occurrence of IL-1β, IL-6, and TNF-α was measured using an IL-1β-, IL-6-, and TNF-α-specific ELISA (Human IL-1β ELISA kit, Invitrogen, ThermoFisher Scientific; Quantikine ELISA Human IL-6, R&D Systems, Minneapolis, MN, USA; and Human TNF-α ELISA kit, Invitrogen; ThermoFisher Scientific) according to the instruction manuals. In brief, the thawed samples were diluted 1:2 (IL-1β) or 1:40 (IL-6) or used pure (TNF-a), and 50 µL (IL-1β), 2.5 µL (IL-6), or 200 µL (TNF-α) was added to the kit’s dilution buffer in a 96-well plate. A standard and the controls were also added to the well plate. Duplicates were performed for every condition. After 2 h of incubation in the dark on a horizontal shaker, the primary antibodies (kit components) were added, and the plate was again incubated and shaken for 2 h. Afterwards, the substrate (kit component) was added, and the color of the samples changed. The reaction was stopped after 15–20 min with 50 µL stop solution (kit component) that triggered a color change to yellow. Finally, the optical density was measured at 540 nm for IL-6 and at 450 nm (IL-1β and TNF-α) with a plate reader. The background control, obtained by replacing samples with PBS, was subtracted, and the samples’ optical density (OD) was determined. The IL-6 concentrations were calculated by a standard series. The ELISAs were verified by the immunoassay control group as well as by MSCs stimulated by lipopolysaccharides (LPSs, Thermo Fisher Scientific).

### 4.5. Real-Time Reverse Transcriptase Polymerase Chain Reaction (Real-Time RT-PCR)

Analyses of mRNA expression were performed at days 0, 5, 10, and 15 of cell culture incubation. A cell monolayer was washed with PBS, lysed with 750 µL Trizol (Life Technologies, Thermo Fisher Scientific), and stored at −80 °C. Then, the total mRNA was isolated with the RNeasy Lipid Tissue Mini Kit (Qiagen, Hilden, Germany) and reverse-transcribed into cDNA using the QuantiTect Reverse Transcription Kit (Qiagen) according to the manufacturer’s protocol. In brief, samples were mixed with 150 µL chloroform (Carl Roth, Karlsruhe, Germany) and centrifuged for 15 min with 14.000 rpm. The RNA phase was mixed with 70% ethanol and transferred to a column. Bound RNA was washed and eluted with 30 µL water, and the RNA concentration was determined by nanodrop (ND-1000, Nanodrop Technologies, Wilmington, DE, USA). RNA (125 ng) was treated with gDNA wipeout buffer (kit component) to destroy genomic DNA. Afterwards, RNA was reverse-transcribed into cDNA by reverse transcriptase diluted in a master mix containing primers and nucleic acids (kit components). After cDNA synthesis, real-time RT-PCR was performed with custom-designed intron-spanning primers (Table 1) using the Quantifast SYBR Green PCR Kit (Qiagen) or the LightCycler Fast Start DNA Master Plus SYBR Green I (Roche Diagnostics GmbH, Mannheim Germany) for the target *RUNX2*. Therefore, cDNA was diluted 1:4 in water and transferred into the master mix containing the buffered enzymes and nucleic acids. Real-time RT-PCR was carried out with 5 min degradation at 95 °C, followed by 40 cycles of 10 s at 95 °C and 30 s at 60 °C. Finally, a melting curve was used to control the sample’s purity. As additional controls, (a) cDNA and (b) reverse transcriptase were omitted during PCR and cDNA syntheses, respectively. The relative expression was calculated by the 2^−ΔΔCT^ method using cycle threshold (Ct) values that were normalized by the reference gene β-2 microglobulin (B2M).

### 4.6. Collagen Type II ELISA

The collagen type II concentration was measured at day 20 of cell culture incubation with a collagen type II ELISA (abbexa, Cambridge, UK). After washing, 220 µL of a papain solution (1 mg/mL diluted in 1 mM ethylenediaminetetraacetic acid (Versene, Gibco)), as well as 500 µL of ice-cold PBS, was added to the cells. Afterwards, the cells were centrifuged for 5 min at 300× *g* and 4 °C, washed with PBS, and homogenized first in a vibration mill (VWR, Darmstadt, Germany) for 1 min and then for 5 min in an ultrasonic bath (Bandelin electronic, Berlin, Germany). Samples were diluted in PBS and transferred to the precoated 96-well plate of the ELISA kit. Further procedures were carried out according to the manufacturer’s operating instructions. In brief, after 1 h of incubation at 37 °C, 100 µL detection reagent A was added, and the plate was incubated again for 1 h at 37 °C. Then, 100 µL detection reagent B was admitted, and the plate was incubated once again for 1 h at 37 °C. After washing, 90 µL of the substrate (kit component) was administered, and the plate was incubated for 30 min at 37 °C. The color changed from colorless to blue. The reaction was stopped by adding 50 µL stop solution and measured at 450 nm using a plate reader. For calculation, the value of the background control (PBS) was subtracted, and the concentration was quantified by the standard curve prepared within the plate with the supplied standard. HEK293 cells were used as a positive control.

### 4.7. Statistical Analyses

SPSS software (version 25, SPSS Institute Inc., Chicago, IL, USA) was used for statistical analyses. First, the values of every data set were tested for normal distribution using the Kolmogorov–Smirnov test. The data from the MTT assay showed a normal distribution. Thus, one-way analysis of variance (ANOVA) was applied to evaluate whether the differences between the group means were statistically significant. Following the ANOVA test, the post hoc test was performed to correct for multiple testing. Since the IL-6, IL-1β, and TNF-α ELISA values were not normally distributed, the non-parametric Kruskal–Wallis test was conducted to compare whether the differences in the 5 independent groups were significant. The values of the relative expression were used for the statistical analysis of real-time RT-PCR. Since the values for TRPV4 and SOX were normally distributed, a paired-samples *t*-test was applied. The values of ACAN, HAPLN1, RUNX2, and COL1A1 were not normally distributed. Therefore, the non-parametric Friedman test for more than two dependent samples was used, since comparing multiple time points leads to the samples being considered related. The Friedman test was followed by Bonferroni correction to avoid statistical errors due to multiple testing. Because of the differences in the median values of the negative control (C) and positive control (CART) for HAPLN1 expression at day 15, we performed the non-parametric Wilcoxon signed-rank test for dependent samples for these two groups. The non-parametric Kruskal–Wallis test followed by the Mann–Whitney U-test was used for the statistical analysis of COL2A1 values because they were not normally distributed and only included one harvesting time point. The values of the collagen type II ELISA were also not normally distributed and were therefore analyzed with a Friedman test followed by Bonferroni correction and a Wilcoxon test. The significance level was set at a value of *p* < 0.05, and the number of experiments and donors was n = 6. Graphs were generated as box plots with SPSS and are described in detail in the legend of Figure 1.

## 5. Conclusions

The results revealed that the cells on LCM and LCMH discs differentiated similarly to the cells in differentiation medium without material contact (CART). The cells on ACM and ACMH discs did not present a significantly higher relative gene expression of chondrogenic targets. In a direct comparison of the material with and without heparin coating, the materials without heparin coating performed better. The results of the IL-6 ELISA showed that the materials did not induce any inflammatory factors. Thus, our approach identified LCM as a suitable material for the generation of cartilage scaffolds using 3D printing methods. LCM does not suppress chondrogenic differentiation, is biodegradable, and enables the mass production of complex biomimetic scaffolds as well as the generation of customized implants with a multi-layered architecture.

## Figures and Tables

**Table 1:** Primer pairs for real-time RT-PCR.

Primer	Sequence	Length (bp)	Accession No.
ACAN	for: CGCCCATCAACAGAGACCTAC rev: CGTGTGGCGAAGAACACCTC	74	NM_001135
B2M	for: TCTCTCTTTCTGGCCTGGAG rev: CAACTTCAATGTCGGATGGA	135	NM_004048.4
COL1A1	for: GCAAGAACCCCAAGGACAAG rev: ATCAGGCGCAGGAAGGTC	130	NM_000088.4
COL2A1	for: GAGCAGCAAGAGCAAGGAG rev: TGTTGGGAGCCAGATTGTC	98	NM_001844
HAPLN1	for: GAGTCTACTTCTTCTGGTGCTG rev: AAACACCTTGGCTTGCTCTG	154	NM_001884.4
RUNX2	for: GGCCTTCAAGGTGGTAGCC rev: ATCGTTACCCGCCATGACA	70	NM_001015051
SOX9	for: AGGTGCTCAAAGGCTACGAC rev: GTAATCCGGGTGGTCCTTCT	281	NM_000346
TRPV4	for: AGGTCATTACGCTCTTCACT rev: CTGAGACGATCACCAGGACA	150	NM_021625

for: forward primer; rev: reverse primer.

## Data Availability

The original contributions presented in this study are included in the Supplementary Materials. Further inquiries can be directed to the corresponding authors.

## Supplementary Materials

The following supporting information can be downloaded at https://www.mdpi.com/article/10.3390/ijms26125837/s1.

## Abbreviations

The following abbreviations are used in this manuscript:

ACAN	Aggrecan
ACM	Polyamid-ε-caprolactone-methacrylate
ACMH	ACM with heparin coating
B2M	Beta-2 microglobulin
BMP2	Bone morphogenetic protein 2
C	Control group with medium without chondrogenic supplements
CART	Control without material but with chondrogenic supplements
COL1A1	Gene encoding for collagen type I alpha chain
DMEM	Dulbecco’s Modified Eagle Medium
DMOG	Dimethyloxalylglycine
FBS	Fetal bovine serum
HAPLN1	Hyaluronan and proteoglycan link protein 1
IL-1β	Interleukin-1β
IL-6	Interleukin-6
LCM	Poly-(D,L)-lactide-ε-caprolactone-methacrylate
LCMH	LCM with heparin coating
MSCs	Mesenchymal stem cells
OA	Osteoarthrosis
RUNX2	Runt-related transcription factor 2
RT-PCR	Reverse transcriptase polymerase chain reaction
SOX9	Relative sex-determining region Y-box transcription factor 9
TGF-β	Tumor growth factor-beta
TNF-α	Tumor necrosis factor-α
TRPV4	Transient receptor potential vanilloid cation channel 4
