# Supplementary material for: Poly-(D,L)-Lactide-ε-Caprolactone-Methacrylate Is a Suitable Scaffold Material for In Vitro Cartilage Regeneration
Source: Int J Mol Sci. 2025 Jun 18;26(12):5837. doi: 10.3390/ijms26125837 (PMC12193312; doi:10.3390/ijms26125837)
Supplement: Supplementary file 1 [file ijms-26-05837-s001.zip › ijms-3603689-supplementary.pdf]

Supplements

Table S1. Detailed data of MTT-Assay

Parameter	Day	Samples	$\bar{x}$ (SD)	x_{max}	x_{min}	p-values
**MTT**<br><br>Units<br>Optical<br>Density<br>(OD)	5	**CART**[0]<br>(n=6)	0.138<br>(0.037)	0.184	0.084	0 vs 1-3 p=1<br>0 vs 3 p<0.001<br>0 vs 4 p=0.009<br>1 vs 0, 2 p=1<br>1 vs 3 p=0.001<br>1 vs 4 p=0.027<br>2 vs 0-1 p=1<br>2 vs 3 p<0.001<br>2 vs 4 p=0.001<br>3 vs 0, 2 p<0.001<br>3 vs 1 p=0.001<br>3 vs 4 p=1
		**LCM**[1]<br>(n=6)	0.130<br>(0.034)	0.173	0.084	
		**LCMH**[2]<br>(n=6)	0.152<br>(0.029)	0.189	0.107	
		**ACM**[3]<br>(n=6)	0.050<br>(0.031)	0.081	-0.006	
		**ACMH**[4]<br>(n=6)	0.073<br>(0.003)	0.076	0.069	

[0]CART: controls without material contact incubated chondrogenic differentation medium; [1]LCM: poly-(D,L)-lactide-ε-caprolactone-methacrylate; [2]LCMH: LCM with heparin coating; [3]ACM: polyamid-ε-caprolactone-methacrylate; [4]ACMH: ACM with heparin coating. Data represent mean ($\bar{x}$), standard deviation of mean (SD), median (x), minimum (x_{min}) and maximum (x_{max}).

Table S2. Detailed data of IL-6 ELISA

Parameter	Day	Samples	$\bar{x}$ (SD)	x_{max}	x_{min}	p-values
**IL-6 ELISA**<br><br>Units<br>pg/ml	5	**CART**<br>(n=6)	88.32<br>(10.99)	104.68	73.76	0.289
		**LCM**<br>(n=6)	97.49<br>(22.51)	126.48	69.00	
		**LCMH**<br>(n=6)	130.1<br>(72.45)	272.76	81.76	
		**ACM**<br>(n=6)	122.78<br>(42.34)	200.84	81.76	
		**ACMH**<br>(n=6)	134.77<br>(68.77)	265.48	72.16	

IL-6: Interleukin-6

Table S3. Detailed data of TNF-α ELISA

Parameter	Day	Samples	$\bar{x}$ (SD)	x_{max}	x_{min}	p-values
**TNF-α ELISA**<br><br>Units Optical Density (OD)	5	**CART** (n=6)	0.049 (0.002)	0.051	0.045	0.698
		**LCM** (n=6)	0.049 (0.003)	0.054	0.045	
		**LCMH** (n=6)	0.051 (0.003)	0.056	0.047	
		**ACM** (n=6)	0.05 (0.004)	0.054	0.045	
		**ACMH** (n=6)	0.052 (0.004)	0.059	0.048	

TNF-α:Tumor necrosis factor-α

Table S4. Detailed data of IL-1β ELISA

Parameter	Day	Samples	$\bar{x}$ (SD)	x_{max}	x_{min}	p-values
**IL-1β ELISA**<br><br>Units Optical Density (OD)	5	**CART** (n=6)	0.056 (0.003)	0.063	0.055	0.291
		**LCM** (n=6)	0.06 (0.004)	0.067	0.057	
		**LCMH** (n=6)	0.057 (0.001)	0.058	0.056	
		**ACM** (n=6)	0.059 (0.002)	0.061	0.056	
		**ACMH** (n=6)	0.059 (0.003)	0.064	0.057	

IL-1β: Interleukin-1β

Table S5. Detailed data of TRPV4 and SOX9 real-time RT-PCR. Results of parameters in real-time reverse transcriptase PCR (real-time RT-PCR) regarding early chondrogenic differentiation in contact with LCM, LCMH, ACM, and ACMH.

Parameter	Day	Samples	$\bar{x}$ (SD)	x_{max}	x_{min}	p-values
Relative gene expression<br>[1]**TRPV4**<br><br>Units<br>$2^{-\Delta\Delta CP}$	5	**C**<br>(n=6)	1.305<br>(0.964)	3.030	0.61	C vs CART<br>p=0.017<br>CART vs ACMH<br>p=0.037<br>all other comparisons<br>p>0.05
		**CART**<br>(n=6)	2.79<br>(0.964)	4.140	0.88	
		**LCM**<br>(n=6)	2.072<br>(1.152)	3.380	0.63	
		**LCMH**<br>(n=6)	1.955<br>(0.819)	3.1	0.78	
		**ACM**<br>(n=6)	2.015<br>(0.8749	2.94	0.66	
		**ACMH**<br>(n=6)	1.827<br>(1.005)	3.58	0.64	
Relative gene expression<br>[2]**SOX9**<br><br>Units<br>$2^{-\Delta\Delta CP}$	5	**C**[0]<br>(n=6)	0.987<br>(0.921)	2.799	0.358	0 vs 1 p=0.025<br>0 vs 2 p=0.029<br>0 vs 3 p=0.031<br>1 vs 2 p=0.003<br>1 vs 3 p=0.04<br>1 vs 4 p=0.012<br>1 vs 5 p=0.027<br>all other comparisons<br>p>0.05
		**CART**[1]<br>(n=6)	3.327<br>(1.634)	4.773	0.993	
		**LCM**[2]<br>(n=6)	2.451<br>(1.37)	3.694	0.745	
		**LCMH**[3]<br>(n=6)	2.374<br>(1.368)	3.797	0.563	
		**ACM**[4]<br>(n=6)	2.137<br>(1.246)	3.681	0.639	
		**ACMH**[5]<br>(n=6)	2.118<br>(1.453)	3.837	0.518	

[1]TRPV4: transient receptor potential vanilloid cation channel-4; [2]SOX9: sex-determining region-Y-box transcription factor-9.

Table S6. Detailed data of ACAN and HAPLN1 real-time RT-PCR. Parameters showing the results of targets for mature chondrogenic differentiation in contact with LCM, LCMH, ACM, and ACMH.

Parameter	Day	Samples	$\bar{x}$ (SD)	x_{max}	x_{min}	p-values
Relative gene expression [1]**ACAN** Units $2^{-\Delta\Delta CP}$	5	**C** (n=6)	54.576 (94.333)	244.722	1.485	p<0.001
		**CART** (n=6)	11.606 (12.542)	30.803	0.576	
		**LCM** (n=6)	6.827 (6.307)	16.056	1.376	
		**LCMH** (n=6)	6.406 (4.696)	12.424	1.288	
		**ACM** (n=6)	2.671 (2.066)	5.856	0.609	
		**ACMH** (n=6)	2.804 (2.248)	6.635	0.664	
Relative gene expression **ACAN** Units $2^{-\Delta\Delta CP}$	10	**C** (n=6)	30.423 (60.865)	154.343	1.602	
		**CART** (n=6)	18.767 (23.637)	63.558	2.056	
		**LCM** (n=6)	14.377 (18.786)	51.268	2.189	
		**LCMH** (n=6)	11.979 (14.315)	37.401	1,84	
		**ACM** (n=6)	6.515 (6.089)	17.63	0.768	
		**ACMH** (n=6)	6.772 (6.621)	18.189	0.681	
Relative gene expression **ACAN** Units $2^{-\Delta\Delta CP}$	15	**C** (n=6)	19.128 (23.124)	58.892	2.888	
		**CART** (n=6)	48.185 (56.485)	147.033	6.105	
		**LCM** (n=6)	33.034 (39.45)	104.691	5.877	
		**LCMH** (n=6)	43.137 (54.857)	140.556	5.657	
		**ACM** (n=6)	9.471 (8.388)	24.761	3.352	
		**ACMH** (n=6)	12.59 (10.64)	28.246	2.078	

Relative gene expression [2]**HAPLN1** Units $2^{-\Delta\Delta CP}$	10	**C** (n=6)	0.693 (0.548)	1.69	0.14	d15: C vs CART p=0.046 all other comparisons p>0.05
		**CART** (n=6)	1.831 (2.255)	6.32	0.28	
		**LCM** (n=6)	2.281 (3.203)	8.69	0.24	
		**LCMH** (n=6)	2.105 (2.782)	7.67	0.32	
		**ACM** (n=6)	2.33 (3.28)	8.97	0.33	
		**ACMH** (n=6)	2.118 (2.987)	8.14	0.29	
Relative gene expression **HAPLN1** Units $2^{-\Delta\Delta CP}$	15	**C** (n=6)	1.045 (1.514)	4.1	0.07	
		**CART** (n=6)	2.818 (4.061)	10.93	0.43	
		**LCM** (n=6)	2.925 (4.514)	12	0.4	
		**LCMH** (n=6)	2.505 (3.919)	10.41	0.34	
		**ACM** (n=6)	1.325 (1.823)	5.01	0.3	
		**ACMH** (n=6)	1.565 (1.887)	5.12	0.24	

[1]ACAN: gene encoding aggrecan; [2]HAPLN1: hyaluronan and proteoglycan link protein

Table S7. Detailed data of RUNX2 and COL1A1 real-time RT-PCR

Parameter	Day	Samples	$\overline{x}$ (SD)	x_{max}	x_{min}	p-values
Relative gene expression [1]**RUNX2** Units $2^{-\Delta\Delta CP}$	5	**C** (n=6)	0,8 (0,528)	1,830	0,380	C vs CART p<0.001 all other comparisons p>0.05
		**CART** (n=6)	3,037 (1,345)	5,13	1,26	
		**LCM** (n=6)	2,327 (1,233)	3,68	0,97	
		**LCMH** (n=6)	2,053 (0,696)	2,91	1,01	
		**ACM** (n=6)	1,697 (0,805)	3,02	1,01	
		**ACMH** (n=6)	1,558 (0,575)	2,62	1,1	
Relative gene expression **RUNX2** Units $2^{-\Delta\Delta CP}$	10	**C** (n=6)	0,5 (0,194)	0,8	0,25	C vs CART p=0.0061 C vs LCM p=0.001 C vs LCMH p=0.039 all other comparisons p>0.05
		**CART** (n=6)	2,693 (2,338)	7,09	0,67	
		**LCM** (n=6)	2,653 (1,753)	5,41	1,02	
		**LCMH** (n=6)	2,215 (1,729)	5,52	0,87	
		**ACM** (n=6)	1,648 (1,117)	3,63	0,75	
		**ACMH** (n=6)	1,655 (1,172)	3,86	0,7	
Relative gene expression **RUNX2** Units $2^{-\Delta\Delta CP}$	15	**C** (n=6)	0,665 (0,415)	1,37	0,22	C vs CART p=0.003 all other comparisons p>0.05
		**CART** (n=6)	3,728 (4,144)	11,84	0,5	
		**LCM** (n=6)	2,773 (3,309)	9,32	0,43	
		**LCMH** (n=6)	2,747 (3,54)	9,78	0,26	
		**ACM** (n=6)	1,628 (1,264)	4,03	0,74	
		**ACMH** (n=6)	2,173 (1,858)	5,43	0,57	

Relative gene expression **[2]COL1A1** Units $2^{-\Delta\Delta CP}$	5	**C** (n=6)	1,231 (0,528)	2,25	0,821	C vs CART p=0.002<br>C vs LCM p=0.039<br>C vs ACM p=0.023<br>all other comparisons p>0.05
		**CART** (n=6)	3,273 (1,584)	6,19	1,647	
		**LCM** (n=6)	2,703 (0,91)	4,257	1,86	
		**LCMH** (n=6)	2,626 (1,028)	3,758	1,315	
		**ACM** (n=6)	2,728 (0,772)	4,042	1,790	
		**ACMH** (n=6)	2,376 (0,796)	3,618	1,424	
Relative gene expression **COL1A1** Units $2^{-\Delta\Delta CP}$	10	**C** (n=6)	0,874 (0,42)	1,464	0,308	C vs CART p=0.045<br>C vs LCM p=0.029<br>C vs LCMH p=0.009<br>C vs ACM p=0.045<br>C vs ACMH p=0.019<br>all other comparisons p>0.05
		**CART** (n=6)	4,57 (2,478)	8,969	1,796	
		**LCM** (n=6)	4,167 (2,429)	8,877	1,86	
		**LCMH** (n=6)	4,315 (2,709)	9,646	1,966	
		**ACM** (n=6)	4,276 (3,111)	10,232	1,886	
		**ACMH** (n=6)	4,522 (3,208)	10,891	1,84	
Relative gene expression **COL1A1** Units $2^{-\Delta\Delta CP}$	15	**C** (n=6)	0,894 (0,505)	1,526	0,178	C vs CART p=0.002<br>C vs LCM p=0.03<br>all other comparisons p>0.05
		**CART** (n=6)	5,601 (5,302)	14,825	1,729	
		**LCM** (n=6)	3,749 (3,497)	10,593	1,189	
		**LCMH** (n=6)	3,595 (4,095)	11,753	0,853	
		**ACM** (n=6)	3,042 (2,433)	7,727	1,218	
		**ACMH** (n=6)	3,483 (2,538)	8,486	1,619	

[1]RUNX2: runt-related transcription factor 2; [2]COL1A1: gene encoding collagen type I

Table S8. Detailed data of COL2A1 real-time RT-PCR

Parameter	Day	Samples	$\bar{x}$ (SEM)	x_{max}	x_{min}	p-values
Relative gene-expression [1]**COL2A1** Units $2^{-\Delta\Delta CP}$	15	**LCM** (n=4)	3.57 (1.61)	7.44	0.5	HEK-293 vs LCM p=0.029 HEK-293 vs LCMH p=0.016 HEK-293 vs ACM p=0.016 HEK-293 vs ACMH p=0.016 all other comparisons p>0.05
		**LCMH** (n=5)	2.74 (1.65)	9.00	0.26	
		**ACM** (n=5)	1.68 (0.7)	4.17	0.11	
		**ACMH** (n=5)	2.33 (0.81)	5.45	0.76	
		**HEK-293** (n=4)	35801.81 (5434.45)	44207	21350	

[1]COL2A1: gene encoding collagen type II

Table S9. Detailed data of Collagen Type II ELISA.

Parameter	Day	Samples	$\bar{x}$ (SD)	x_{max}	x_{min}	p-values
**Collagen Type II ELISA** Units pg/ml	20	**C** (n=6)	1113.076 (611.09)	1679.612	465.516	C vs LCM, LCMH, ACM p=0.043 LCMH vs ACMH p=0.043 all other comparisons p>0.05
		**CART** (n=6)	4045.831 (3699.984)	9720.988	724.964	
		**LCM** (n=6)	4399.546 (1835.876)	6732.656	2062.140	
		**LCMH** (n=6)	4330.273 (2706.465)	7659.324	1579.824	
		**ACM** (n=6)	3891.752 (1748.732)	2367.508	6853.724	
		**ACMH** (n=6)	3586.238 (2837.217)	7143.604	868.92	
